# Immunohistochemical Evaluation of Spinal Cord Injuries Treated with Amniotic Membrane

**DOI:** 10.1021/acsomega.5c07369

**Published:** 2026-01-27

**Authors:** Leonardo B. de Lima, Débora C. C. Correia, Luciana B. Sant’anna, Emilia A. L. S. Arisawa

**Affiliations:** Biostimulation and Tissue Repair Laboratory, Development and Research Institute (IP&D), 67655 Universidade Do Vale Do Paraíba- UNIVAP, Av. Shishima Hifumi, 2911, Urbanova, São José dos Campos, São Paulo 12244-000, Brazil

## Abstract

Spinal cord injury (SCI) severely disrupts central nervous system (CNS) function by interrupting sensory and motor signal transmission, often resulting in permanent deficits due to the formation of a glial scar. Although the amniotic membrane (AM) is derived from the human placenta and is a promising biomaterial, its efficacy in treating SCI remains unexplored. This study investigates the therapeutic potential of AM fragments in a surgically induced acute SCI model in rats, focusing on preserving tissue integrity and modulating astrocyte distribution and reactivity. SCI was experimentally induced by a drop-weight mini-guillotine model in rats, which were subsequently allocated into three groups: Control (C), Injury (I), and Amniotic Membrane (AM), where a 4 cm^2^ AM fragment was applied over the lesion. Animals were euthanized after 28 days for histological and immunohistochemical analysis of the T9-T10 region, specifically to assess Glial Fibrillary Acidic Protein (GFAP) expression and identify reactive astrocytes. The application of AM significantly preserved nervous tissue structure. The cystic cavity area in the AM group (9.00 ± 7.65) was drastically lower than in the Injury group (41.80 ± 11.30). Crucially, the AM fragments attenuated the progression of nervous tissue degeneration, limiting cavitation and glial scar formation while reducing astrocytic reactivity. These findings establish AM as a viable and effective scaffold for acute SCI treatment.

## Graphical abstract

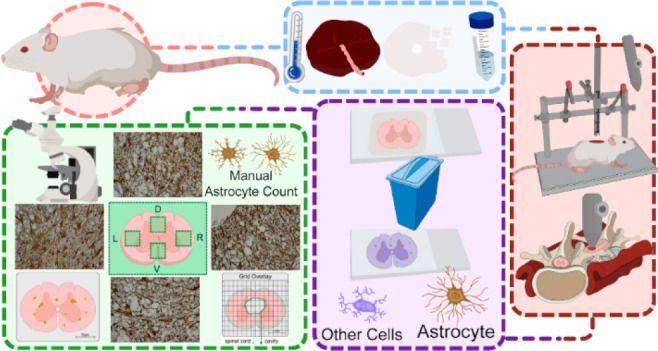

## Introduction

The spinal cord (SC) serves as a fundamental communication channel within the central nervous system (CNS), regulating both sensory and motor functions. It receives signals from and transmits signals to the peripheral nervous system (PNS).^1^ Spinal cord injuries (SCI) are characterized by partial or complete interruption of these signals, which significantly impacts the quality of life of affected individuals.^2^ The severity and resulting disability of SCI are directly related to the region and intensity of the injury. These injuries are commonly associated with motor vehicle accidents, domestic or occupational accidents, violence, sports, infections, tumors, and herniated discs. SCI predominantly affects men of working age, and effective public health preventive actions remain insufficient.^3–5^ SCI pathology is classically divided into two phases. The primary phase is caused by direct aggression to the nervous tissue, resulting in cellular destruction, vascular and axonal rupture, and structural damage. The secondary phase is consequent to the ensuing inflammatory response, which includes edema formation, release of pro-inflammatory mediators and reactive oxygen species, recruitment of immune cells, and subsequent cellular degeneration and necrosis.^6–9^ Endogenous mechanisms associated with impaired biochemical changes in the microenvironment favor the progression of tissue damage, thereby hindering homeostasis. Microscopically, SCI induces microcavitation and macrocavitation, cellular necrosis, architectural changes, vascular rupture, neuronal loss, axonal rupture, and glial attraction, primarily involving astrocytes and microglia.^10–13^ Histological techniques can identify cell death and the subsequent formation of glial scars in the nervous tissue. Immunohistochemistry, based on the antigen–antibody complex, is used to detect specific structures with high sensitivity. Astrocytes, the glial cells predominantly associated with glial scarring, present glial fibrillary acidic protein (GFAP) in their intermediate filaments, which is commonly employed as a specific marker.^14^ Astrocytes belong to the neural stroma, providing structural support and nutrition, and acting directly on blood vessels. They are classified as protoplasmic astrocytes (gray matter) and fibrous astrocytes (white matter). Postinjury astrocytic reactivity leads to significant changes in the tissue microenvironment, including the loss of parenchymal cells and their replacement by stromal cells, ultimately resulting in the formation of a glial scar. This scar acts as a physical and chemical barrier, severely impairing tissue repair.^15–18^ The pathophysiological complexity of SCI results in limited and inefficient treatment protocols, leading to severe and irreversible sequelae, including permanent motor and sensory loss.^19^

Current research efforts are directed toward controlling the inflammatory process and secondary complications following SCI. The amniotic membrane (AM) is emerging as a promising biomaterial in tissue therapy, given its wide availability, ethical viability, and demonstrated applicability in experimental^20^ and clinical studies.^21^ Obtained from the human placenta, AM has shown efficacy in several therapeutic strategies, including the treatment of burns,^22^ tendon injuries,^23^ liver fibrosis,^24^ spinal cord injury,^25^ diabetic foot ulcers,^21^ and ophthalmological conditions.^26^ AM offers several therapeutic advantages, such as anti-inflammatory, antinecrotic, antifibrotic, and antimicrobial properties, which are associated with an immunomodulatory response and its ability to act as a cellular scaffold.^27–29^

Considering these beneficial aspects, this study aimed to evaluate the efficacy of AM in treating surgically induced spinal cord injuries, focusing on tissue integrity and the distribution and reactivity of astrocytes immunostained for the glial fibrillary acidic protein (GFAP) across different anatomical regions of the spinal cord.

## Materials and Methods

This study was approved by the ethics committees (A17/CEUA/2014; 063/2011-PH/CEP) and used 15 male Wistar rats (60 days, 230 ± 20 g), which were divided into Control (C), Injury (I), and Amniotic Membrane (AM) groups. The use of Wistar rats is a standard and widely accepted practice in SCI research due to the strain’s standardized genetic background, well-established historical data, and suitability for behavioral assessment, ensuring high reproducibility and comparability across studies. The specific choice to use male rats aligns with both the need for rigorous experimental control and the epidemiological reality of SCI incidence. Our choice directly reflects the patient demographic most affected by SCI, as highlighted by the epidemiological data from Brazil.^30^ Approximately 82.9% of those affected by SCI are male, and the victims are predominantly young men, with 60% falling between the ages of 10 and 30 years old, with an average age of 26.55 years. The selection of young adult Wistar male rats thus ensures that our experimental model is both scientifically controlled and highly relevant to the most prevalent population targeted by future SCI therapies.^31^

The amniotic membrane (AM) was obtained from human placentas following term cesarean sections. Donor eligibility was confirmed by seronegativity for HIV, hepatitis B/C, and syphilis. Aseptic processing involved the careful detachment of the AM from the chorion, followed by extensive washing with a physiological solution. This solution was supplemented with antibiotics (100 U/mL of penicillin, 100 μg/mL of streptomycin) and an antifungal (0.5 μg/mL of amphotericin B) until the membrane achieved a transparent appearance. Immediately following processing, the amniotic membrane was sectioned into 4 cm^2^ fragments. The fragments were marked to distinguish the epithelial and mesenchymal sides, stored in Dulbecco’s Modified Eagle Medium (DMEM) at ambient temperature (4 °C), and utilized within 24 h.

Surgery commenced with anesthesia administered intramuscularly: ketamine (50 mg/kg), xylazine (5 mg/kg), and tramadol (10 mg/kg). Following dorsal trichotomy and a midline longitudinal incision, a laminectomy was performed at the T9-T10 vertebral level to expose the spinal cord. Experimental groups were treated as follows:

Control (C) group: surgery terminated after spinal column exposure; no laminectomy or contusion was performed.
Injury (I) group: a controlled contusion injury was induced on the exposed spinal cord using a custom mini-guillotine, with a 10 g weight dropped from a height of 25 mm, and removed from SC after 15 s.
Amniotic membrane (AM) group: immediately following the contusion, a 4 cm^2^ fragment of Amniotic Membrane (AM) was placed to cover the injured area.

The surgical site was closed in layers via soft tissue suture.

Postoperative Monitoring and Management- Animals were housed individually for the first 5 days postsurgery. Prophylactic antibiotic treatment consisted of enrofloxacin (5 mg/kg, administered every 12 h). Pain was managed with tramadol (10 mg/kg, administered every 12 h). After the initial 5-day period, animals were relocated and housed together in cages containing five animals each.

On the twenty-eight postinjury day, all animals were euthanized via a lethal overdose of an anesthetic cocktail (ketamine 150 mg/kg, xylazine 15 mg/kg). The T9-T10 spinal cord segment was immediately excised and subjected to rigorous histological processing. Samples were first fixed in 10% buffered formalin for 24 h, followed by transfer to 70% ethanol before proceeding to tissue processing.

Histological preparation - to prepare the bone structure for sectioning, samples underwent decalcification in EDTA for 30 days, with the solution refreshed every 48 h. Subsequently, the samples were dehydrated using graded alcohols, diaphanized in xylene, and embedded in Paraplast wax. Sections were obtained at a thickness of 4 μm using a Leica RM2245 microtome and mounted onto polylysine-coated slides for subsequent immunohistochemically and histochemical analysis.

The full procedural details, including the SCI induction and the immunohistochemistry (IHC) protocol using the GFAP-Abcam biomarker for astrocytes, are outlined in Figure 1.

**Figure 1:**
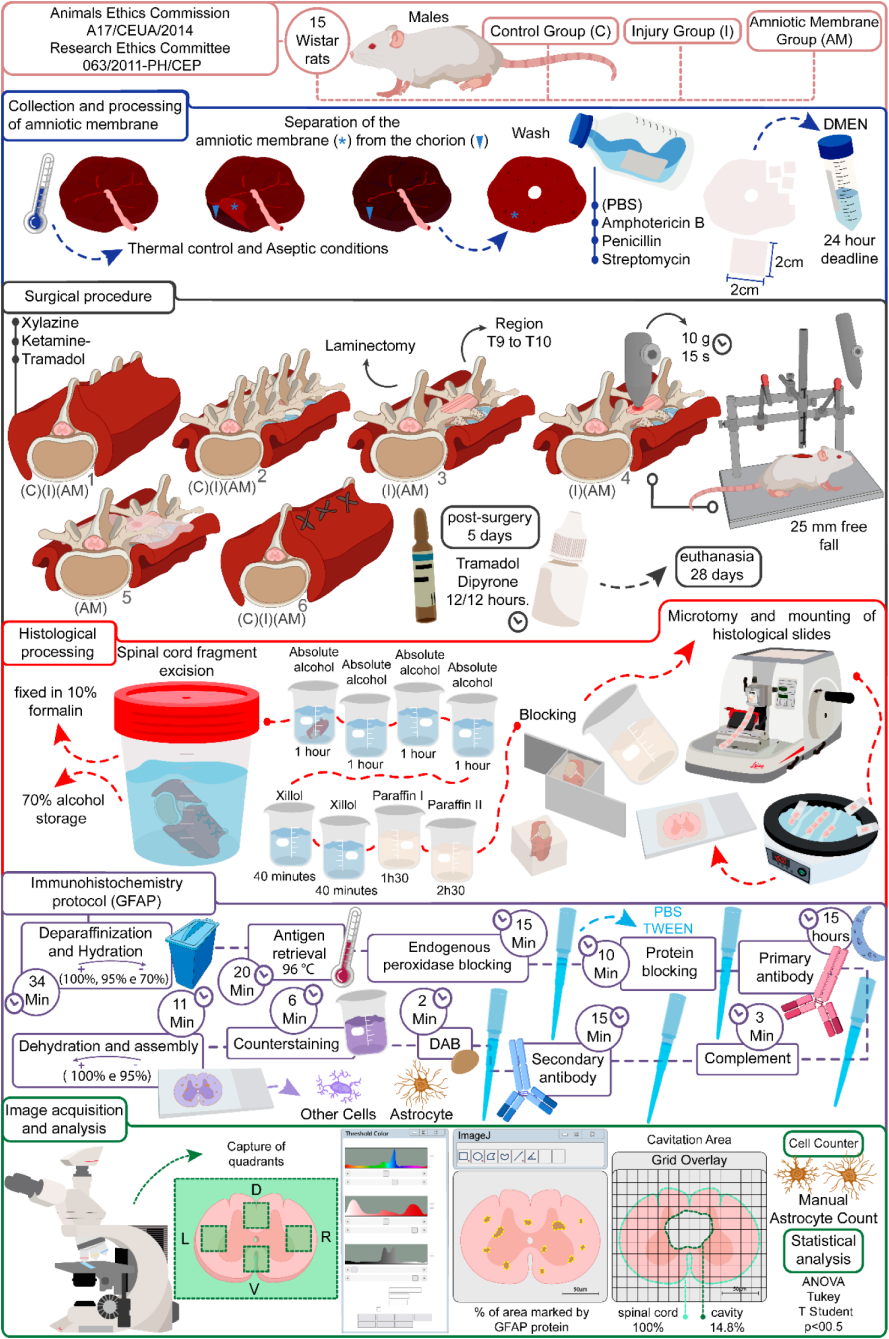
Schematic representation of the experimental protocol for representative diagram of the experimental protocol for qualitative and quantitative astrocytes evaluation with amniotic membrane treatment in spinal cord injury. This figure illustrates the timeline and key procedures for the experimental groups analyzed in this study.

Sections encompassing the session epicenter and adjacent slides from the epicenter and adjacent regions of the LM were processed for the IHC. Deparaffinization was conducted at 57 °C for 30 min, rehydrated through xylene and graded alcohols, and washed in PBS-Tween 20. Antigen retrieval was performed in citrate buffer (10 mM, pH 6.0) at 96 °C for 20 min. Endogenous peroxidase was blocked with hydrogen peroxide (3%, 15 min). Slides were incubated with protein blocking solution (EXPOSE Mouse and Rabbit Specific HRP/DAB Detection IHC kit, Abcam, ab80436) for 10 min to block nonspecific sites. The primary monoclonal anti-GFAP antibody (ab10062, Abcam, clone GF5) was incubated at a dilution of 1:500 at 4 °C for 14–16 h. After washing, specific reagents from the Abcam kit and the antirabbit secondary antibody were applied sequentially. The chromogenic substrate DAB revealed the immunohistochemical reaction within 2 min. The slides were counterstained with Harris hematoxylin for 35 s, dehydrated, immersed in xylene, and mounted with Permount.

Tissue architecture, astrocyte quantity, and GFAP immunoexpression were assessed qualitatively and quantitatively via digital image analysis. Images corresponding to the epicenter and its dorsal, ventral, right, and left quadrants were captured using a Leica DM2500 microscope equipped with a digital camera (LEICA DFC 425). Images were digitized (1024 × 768 pixels, 24 bits/pixel) and magnified at 400×. The Image-J software (v. 1.54, National Institute of Health, USA) was used for histomorphometry analysis. Astrocytic reactivity was assessed by manually counting the numerical density of hypertrophic/gemistocytic astrocytes in representative histological sections of each experimental group (C, I, and AM), using the Cell Counter software. To analyze the homogeneity of the response, the count was segmented and quantified across four quadrants (dorsal, ventral, right, and left) within each group. The percentage of cavitation area and of gray matter area, both relative to the total tissue area, were determined by the point counting method (stereology). This involved superimposing a grid of 180 points onto the micrographs of the sections and counting the points incident on the respective areas. The predominant regional location of the cystic cavity was identified by the percentage of cavitation area in each quadrant. The expression of Glial Fibrillary Acidic Protein (GFAP), a marker for astroglia, was quantified in immunohistochemically treated sections using the threshold method (intensity threshold) in image analysis software. The result was expressed as a percentage of the labeled area. Similar to the astrocytic to evaluate the distribution of expression.

The data were analyzed statistically using the Python programming language within the Google Colab environment. All quantitative data were statistically analyzed with a significance level of *p* < 0.05. To compare the variables (cavitation, GFAP, total astrocyte count, and gray area) among the three groups (C, I, and AM), Analysis of Variance (ANOVA) was used, followed by Tukey’s post hoc test for multiple comparisons. To evaluate the regional location of the cystic cavity, the paired Student’s *t*-test was used to compare the percentage of cavitation area between homologous regions (Right vs Left Side and Ventral vs Dorsal Region) within each group separately. Graphs were plotted using Google Colab programming system.

## Results

Immunohistochemical analysis of the Glial Fibrillary Acidic Protein (GFAP) enabled the identification of astrocytic morphology and the quantification of its expression in the ventral, dorsal, right, and left regions of the spinal cord, revealing distinct patterns in each experimental group.

The Control group showed preserved tissue architecture and the absence of cavities (Figure 2A). The group (I) presented a large cystic cavity and multiple microcavities. In the I group, the loss of tissue architectural integrity was evident, compromising the white matter, gray matter, and the central canal (Figure 2B). The AM group tissue architecture integrity was largely maintained, with well-defined white and gray matter, and the central canal showing only discrete changes. Cavities of reduced extension were visible, and these microcavities were less evident than those observed in the I group (Figure 2C).

**Figure 2:**
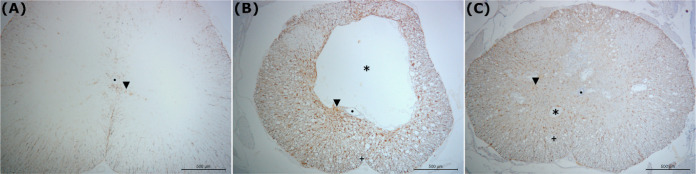
Qualitative immunohistochemical analysis of GFAP staining in the Control (A), Injury (B), and Amniotic Membrane (C) groups. The images illustrate the preservation effects of AM against SCI-induced pathology. Magnification is 5X (scale bar: 500 μm). GFAP-stained astrocytes (inverted triangle), cavitation area (cystic cavity) (asterisk), central canal (dot), and Microcavities (plus). Immunostained by (GFAP).

Qualitatively, the Control (C) group exhibited minimal GFAP immunoexpression and normal astrocytic morphology, nonreactive (Figure 3C-V, C-R, C-D, C-L). This contrasted sharply with the Injury (I) group, which showed intense GFAP immunostaining and numerous hypertrophic/gemistocytic astrocytes characterized by increased volume and extended cytoplasmic processes, observed predominantly around the cavities (Figure 3I-V, I-R, I-D, I-L). In the Amniotic Membrane (AM) group, there was a reduction in GFAP immunostaining and a smaller number of hypertrophic astrocytes compared to the I group. These cells showed reduced morphology and less extensive cellular processes. The GFAP–stained astrocytes were numerous, displaying reduced size and reactivity, and were distributed diffusely throughout the medullary tissue (Figure 3AM-V, AM-R, AM-D, AM-L).

**Figure 3:**
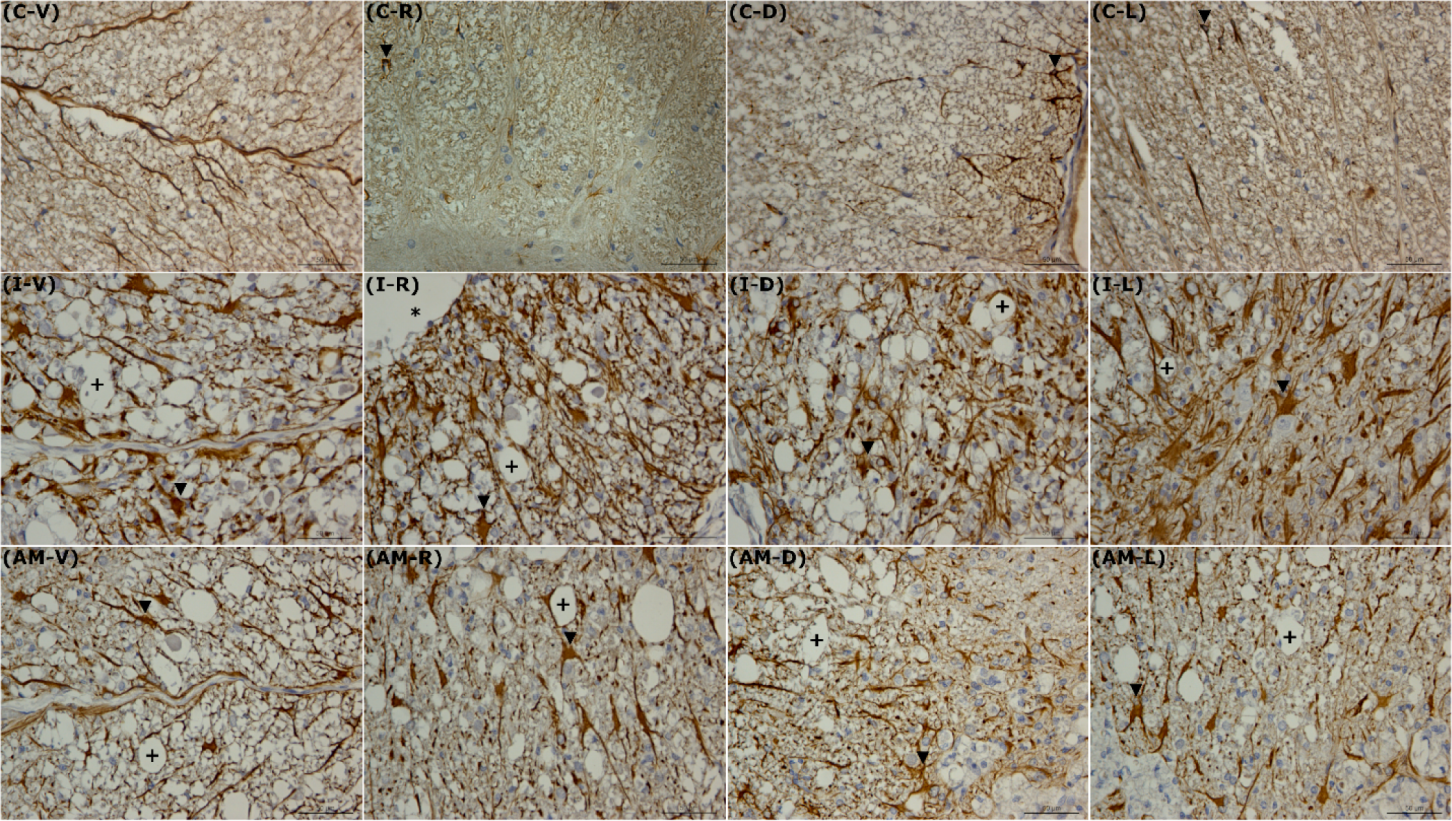
Detailed quadrant immunohistochemical analysis of astrogliosis and cavitation post-SCI. Representative photomicrographs, magnification (scale bar: 50 μm). The figure provides a detailed high-magnification visualization of the cellular pathology, contrasting the baseline condition with the severe injury and the AM-treated outcomes. Symbols indicate: immunostained astrocytes (inverted triangle), cystic cavities (asterisk), and micro cavities (plus). Control (C): Ventral (C-V), Right (C-R), Dorsal (C–D), Left (C-L). Injury (I): Ventral (I-V), Right (I-R), Dorsal (I-D), Left (I-L). Amniotic Membrane (AM): Ventral (AM-V), Right (AM-R), Dorsal (AM-D), Left (AM-L). Immunostained by (GFAP).

Quantitatively, the Control group (C) registers no cavitation (0.00% ± 0.00% cystic area), whereas the Injury group (I) exhibited 41.80% ± 11.30% of cavitation area, indicating extensive tissue destruction. In contrast, the Amniotic Membrane group (AM) demonstrated an area of 9.00% ± 7.65% cystic cavities. Although the AM values showed no statistical significance compared to the Control group, the Amniotic Membrane group values were significantly lower than the I group (C: 0.00% ± 0.00% vs AM: 9.00% ± 7.65%, *p* > 0.05) and (AM: 9.00% ± 7.65% vs I: 41.80 ± 11.30%, *p* < 0.05) (Figure 4A).

**Figure 4:**
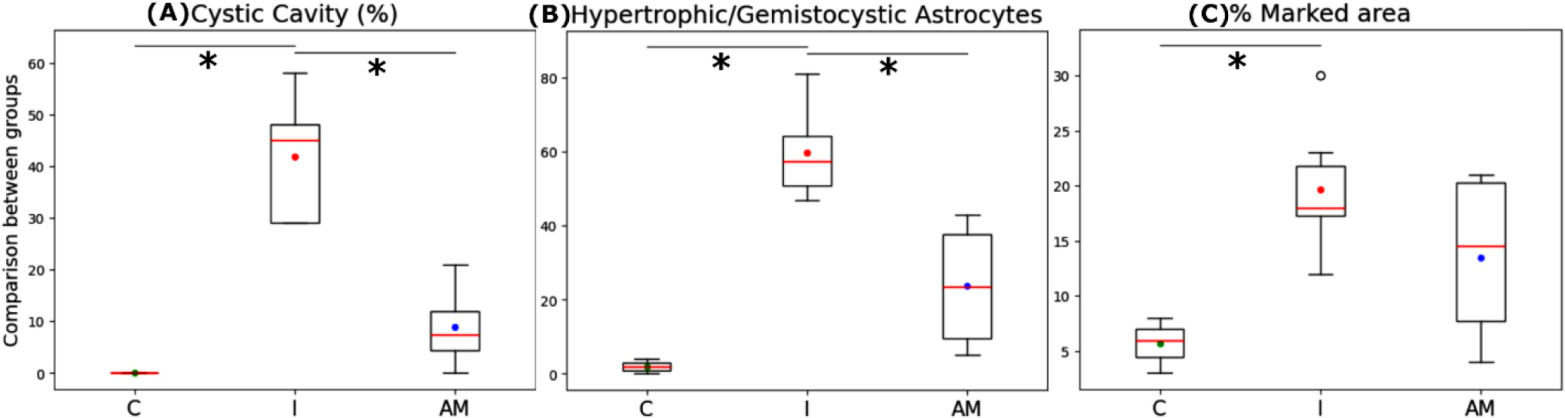
Quantitative assessment of spinal cord injury and Therapeutic Efficacy. (A) Percentage of the cystic cavity area relative to the total tissue area. (B) Quantification of hypertrophic/gemistocytic astrocytes (cell/section). (C) Percentage of tissue area exhibiting GFAP immunostaining (glial scar area) relative to the total tissue area. Data compares Control (C), Injury (I), and Amniotic Membrane (AM) groups. Note: statistically significant differences were observed between the (I) and (AM) groups across cystic cavity and astrocytes parameters are indicated in asterisk (*p* < 0.05).

Analysis of tissue components revealed the postinjury gray matter susceptibility. Group I presented an 87% reduction in gray matter area compared to the Control group (C: 45.00% ± 1.00% vs I: 5.80% ± 2.79%, *p* < 0.05). The AM group significantly preserves gray matter, demonstrating a 47% loss compared to the Control group (C: 45.00% ± 1.00% vs AM: 21.25% ± 5.89%, *p* < 0.05), indicating therapeutic efficacy. The white matter area, conversely, showed no statistically significant difference across the groups (Figure 5).

**Figure 5:**
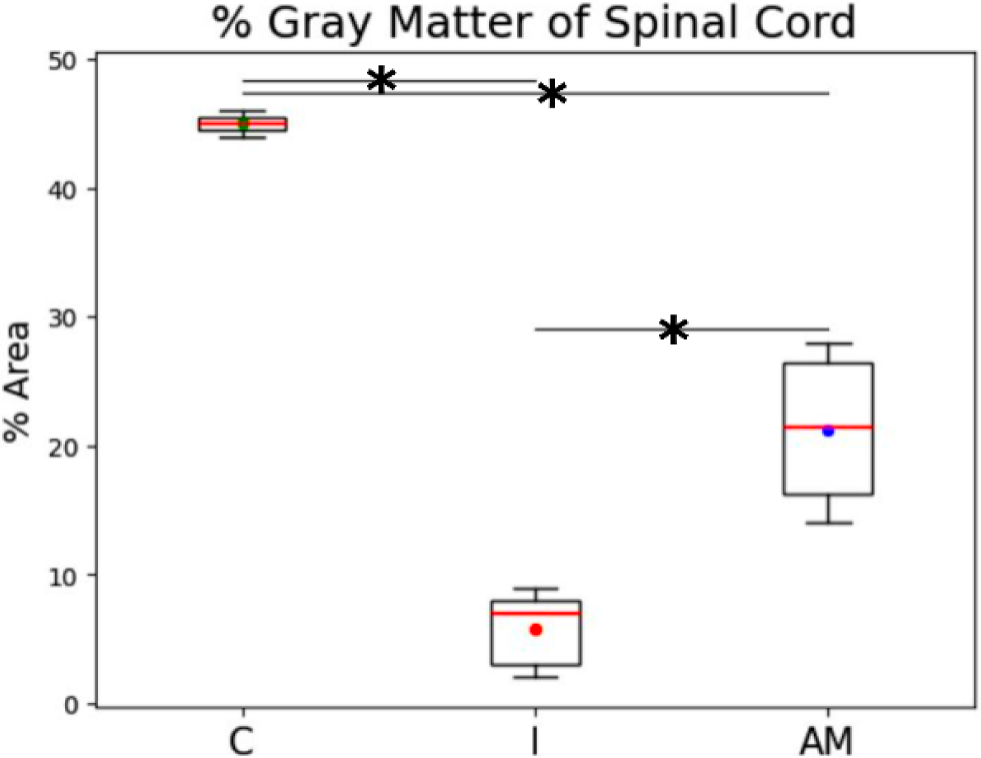
Percentage of gray matter area in the spinal cord. Comparison of the gray matter area among the Control (C), Injury (I), and Amniotic Membrane (AM) groups. Values are expressed as mean ± standard deviation. Asterisk (*) indicates a statistically significant difference (*p* < 0.05) for the comparison between the indicated groups.

Anatomically, the cystic cavity in the (I) group was predominantly positioned in the dorsal region, close to the SCI region. This dorsal positioning showed a statistically significant difference compared to the ventral region (D: 67.20 ± 9.30% vs V: 32.80 ± 9.30%, *p* < 0.05). Furthermore, no statistical difference was observed between the right and left sides (*p* > 0.05). The AM group also showed no statistical difference in the position of the cystic cavity when comparing the right versus left and dorsal versus ventral quadrants (*p* > 0.05). The Control group (C) showed no cavitation area (Figure 6).

**Figure 6:**
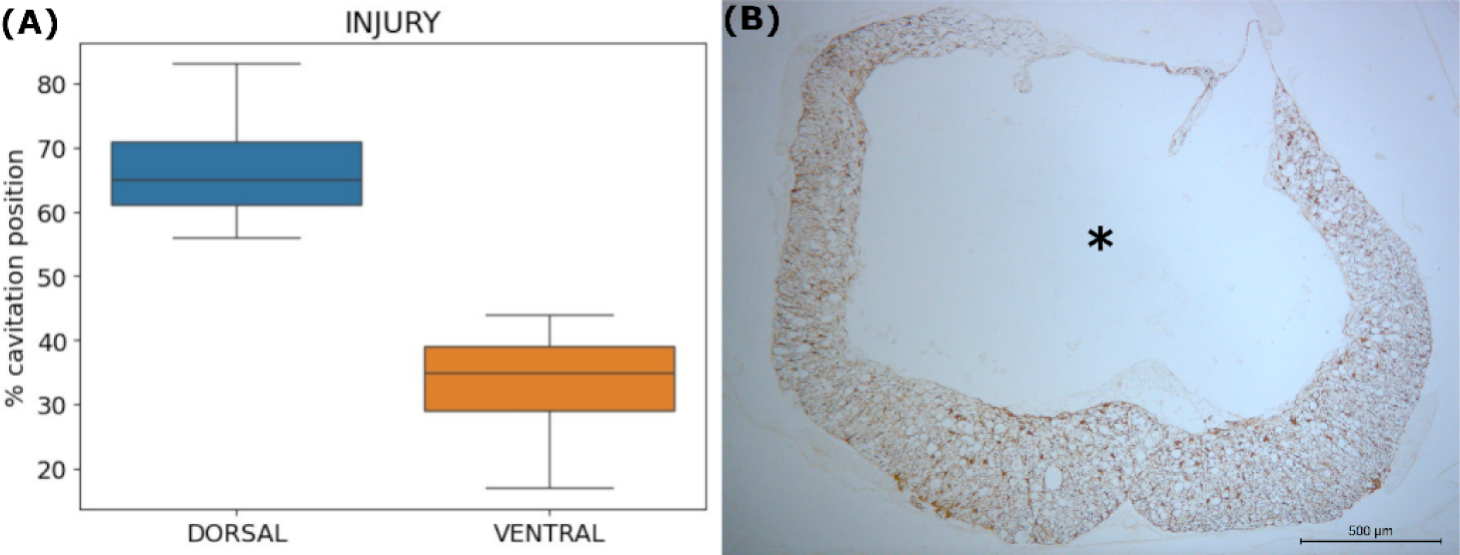
Anatomical analysis of the positioning of the spinal cord cystic cavity in the injury group and corresponding histological photomicrograph. (A) Prevalence of the percentage of cystic cavity area in the spinal cord, comparing the dorsal and ventral quadrants in the untreated group. The data show a statistically significant difference between the quadrants (*p* < 0.05). (B) Representative photomicrograph of the injury site, demonstrating the formation of the cystic cavity (asterisk). Scale bar: 500 μm. Immunostained by GFAP.

The quantification of hypertrophic/gemistocytic astrocytes revealed that the Control (C) group presented an average of 2.00 ± 1.63 astrocytes with normal morphology, showing no significant regional differences (Figure 7A). In contrast, the injury (I) group exhibited a statistically significant increase in hypertrophic/gemistocytic astrocytes (59.67 ± 11.46), with a uniform distribution across all regions (Figure 7B). The amniotic membrane (AM) group, in turn, demonstrated an average of 23.75 ± 16.08 hypertrophic/gemistocytic astrocytes, which represents a 60.2% reduction in astrocytic reactivity and gliosis compared to the I group. Furthermore, while the number of astrocytes in the AM group was not statistically different compared to the C group (C: 2.00 ± 1.63 vs AM: 23.75 ± 16.08, *p* > 0.05), it showed a statistically significant difference compared to the I group (AM: 23.75 ± 16.08 vs I: 59.67 ± 11.46, *p* < 0.05), as illustrated in Figure 4B. The astrocyte counts in the AM group also lacked statistically significant differences between regions (Figure 7C).

**Figure 7:**
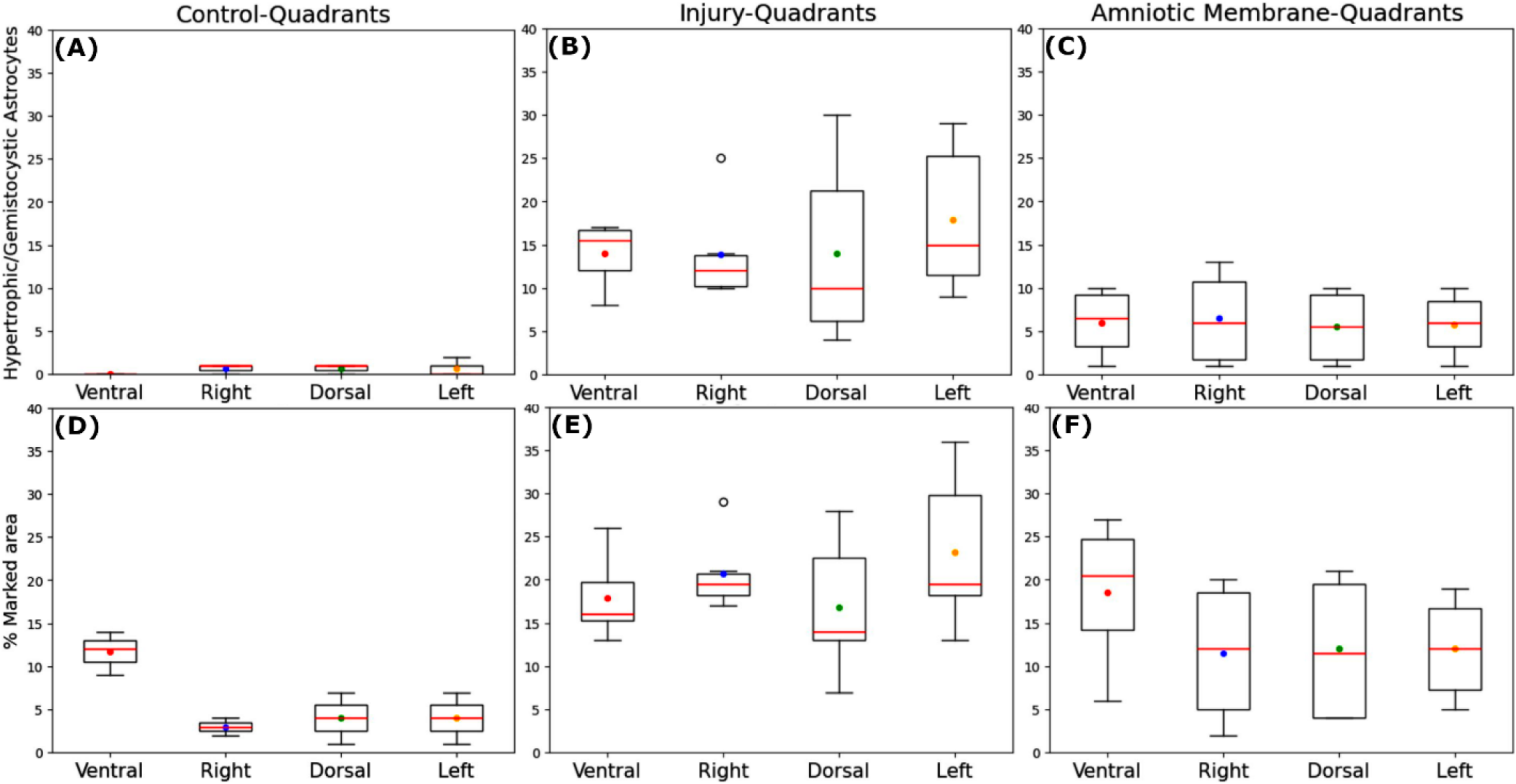
Regional quantification of reactive astrogliosis and lesion distribution. Graphs illustrating the hypertrophic/gemistocytic astrocytes across the ventral, right, dorsal, and left quadrants for: (A) Control, (B) Injury, and (C) Amniotic Membrane. Additionally, the figure presents the percentage distribution of the total pathological area across all quadrants for the Control (D), Injury (E), and Amniotic Membrane (F) groups. Overall statistical comparison across the three groups was (*p* < 0.05).

Quantitative analysis of the area marked by the GFAP protein relative to the total spinal cord tissue area revealed that the Control group (C) presented 5.67 ± 2.05% labeling. This labeling was greater in the ventral region compared to the other regions (*p* < 0.05) (Figure 7D). The Injury group (I), with 41.80% tissue loss, exhibited an average GFAP-marked area of 19.67 ± 5.62%, showing no regional differences (*p* > 0.05) (Figure 7E). The AM group showed GFAP immunostaining (Figure 7F) of 13.50 ± 7.23%, without a statistical difference between regions (*p* > 0.05). Comparing the percentage of GFAP protein labeling in the total tissue area with the control group, no statistically significant difference was found between the AM group and the C group (C: 5.67 ± 2.05% vs AM: 13.50 ± 7.23%, *p* > 0.05). Similarly, no statistically significant difference was found between the AM group and the I group (AM: 13.50 ± 7.23% vs I: 19.67 ± 5.62%, *p* > 0.05) (Figure 4C). Although the group I exhibited a reduced total tissue area compared to the AM group, attributable to cavitation, the expression of the GFAP protein in absolute numbers was numerically higher than that observed in the AM group, without statistical significance.

The immunohistochemical analysis of GFAP established clear patterns among the experimental groups. The Control group (C) exhibited low, nonreactive astrocytic presence (2.00 ± 1.63 astrocytes) and preserved tissue architecture, with an absence of cavitation and 5.67 ± 2.05% of the area stained by the GFAP protein. In sharp contrast, the injury (I) group presented intense astrogliosis (59.67 ± 11.46 hypertrophic astrocytes) and extensive tissue destruction, which culminated in 41.80% ± 11.30% cavitation, an 87% reduction of gray matter, and 19.67 ± 5.62% of the area stained by the GFAP protein. The AM group demonstrated significant attenuation of this pathology, decreasing gliosis by 60.2%, and reducing the cavitation area to only 9.00% ± 7.65%, significantly lower than the I group. Collectively, these findings indicate that spinal injury causes severe reactive gliosis and tissue loss, and intervention with the amniotic membrane reduced the lesion size, preserved architectural integrity, and minimized post-trauma astrocytic reactivity.

## Discussion

Immunohistochemical studies investigating the efficacy of the amniotic membrane (AM) in treating spinal cord injuries (SCI) are highly limited. Consequently, the results presented here, which rigorously evaluated central nervous tissue integrity and astrocytic distribution/reactivity following AM application in groups, are pioneering in this field.

The Control group (C), subjected only to surgical exposure, confirmed a baseline of healthy nervous tissue architecture characterized by nonreactive astrocytes and negligible GFAP staining. In stark contrast, the Injury group (I) demonstrated massive pathology, including extensive areas of cavitation and necrosis enveloped by severe astrogliosis, identified by the presence of numerous hypertrophic astrocytes. The glial reaction in the I group significantly exceeded that observed in both the C and the AM groups.

Interestingly, GFAP immunoreactivity did not exhibit a pronounced dorsal predominance, despite the primary contusion insult being delivered to this region. This finding suggests a rapid, widespread inflammatory response that transcended the immediate injury site.

Astrocytic cell proliferation is a critical component of the endogenous tissue repair response following spinal cord injury (SCI).^2^ In cases of severe central nervous system trauma involving extensive destruction of parenchymal and stromal cells, astrocytes consolidate into the glial scar—a robust physical and chemical barrier.^12,32–34^ While this barrier is crucial for containing the expansion of the initial lesion and maintaining tissue homeostasis, it presents a significant impediment to long-term recovery by actively inhibiting axonal function and reconnection. Our results strongly corroborate this established paradox. Specifically, our data align with the findings of Okada et al.,^33^ who characterized reactive astrocyte activity and glial scar formation as detrimental elements of secondary injury that severely hinder the restoration of the spinal cord microenvironment.

Studies have consistently suggested that glial scar formation is fundamentally mediated by astrocyte proliferation, which is driven by specific microenvironmental signaling within the spinal cord. This proliferation leads to a heterogeneous scar, characterized by significant morphological variations.^12,15,34^ This pattern is similar to the peripheral arrangement of reactive astrocytes observed around the cystic cavities in our Injury group (I). The GFAP immunohistochemical marker proved essential for visualizing and quantifying these changes in astrocyte morphology, distribution, and reactivity, underscoring its relevance for understanding CNS neuropathology.^14^

The consequences of spinal cord injury are known to vary based on several factors, including the location and intensity of the injury. Although the contusion in our model was delivered dorsally, the astrocyte reactivity and GFAP immunoexpression were uniformly distributed across all quadrants in both the I and AM groups. This uniform impact suggests that the injury’s destructive cascade equally affects both ventral motor neurons and dorsal sensory neurons, irrespective of the primary impact site.

Our results demonstrated the therapeutic efficacy of the Amniotic Membrane fragment. The immediate application of AM significantly reduced the area of tissue necrosis and maintained the overall tissue architecture compared to the I group. This strong preservation effect suggests a significant modulatory role of this biomaterial in controlling astrocytic reactivity and the subsequent formation of the glial scar.

Isolated therapies such as stem cells, chemical mediators, growth factors, and the use of synthetic scaffolds loaded with drugs and bioactive molecules have shown benefits in SCI treatment. However, these approaches often involve time-consuming, expensive, and complex processes, which limit their broad accessibility and clinical transposition.^9,35,36^ According to Coassin et al.^37^ and Zhou et al.^38^ AM contains and secretes various immunomodulatory factors, including anti-inflammatory agents, chemical mediators, and growth factors, while also acting as a scaffold to restore the microenvironment of the injured spinal cord.

The findings of this study suggest that AM prevented the expansion of the secondary lesion by limiting the progression of spinal cord injury in nervous tissue. This property, which involves reduction of astrocytic reactivity and overall tissue damage, suggests an anti-inflammatory potential. Consistent with the antifibrotic effects of AM observed in hepatic fibrosis by Alves et al.,^27^ our immediate application of AM fragment after SCI resulted in a significant reduction of the immunolabeled glial scar. This outcome suggests that AM-mediated neuromodulation mitigates astrocyte-driven fibrosis, a process that appears instrumental in facilitating functional recovery.

The Amniotic Membrane represents a significant advance for SCI treatment, given its low collection, processing, and storage costs, wide availability as a discarded postpartum material, and minimal risk of immune response. Our findings on astrocyte plasticity strongly support this therapeutic potential. Consistent with the literature, which shows that astrocytes alternate between nonreactive and reactive forms,^39^ the Control group (C) exhibited no reactive astrocytes, whereas the Injury group displayed intense astrocyte reactivity. Conversely, the AM group exhibited reduced astrocyte reactivity and quantity. These distinct morphological and cellular characteristics strongly suggest that AM treatment effectively modulated the hostile microenvironment following SCI.

The greatest challenge in SCI treatment is the lack of a safe method to eliminate the physical and chemical barriers posed by the glial scar. Nevertheless, the evidence that AM possesses the potential to degrade collagen, a key factor in astrocytic adhesion and scar formation,^27,39,40^ suggests a mechanism that transcends its antinecrotic, anti-inflammatory, immunomodulatory, and supportive effects, thereby establishing a clear antifibrotic action.^41–45^ Early intervention is critical to reducing long-term sequelae; however, current protocols often proceed, despite glial scar formation. The limited effectiveness of these protocols, frequently resulting in motor deficits, stems from the lack of research focused on reducing or interrupting glial scar formation by decreasing astrocytic reactivity. Our study addresses this critical gap by demonstrating a promising reduction of astrocytic reactivity and cavitation formation. This outcome minimizes secondary injury progression and glial scarring, creating an environment that favors tissue homeostasis and potential motor recovery, all while utilizing a low-cost, widely available biomaterial that maintains the necessary barrier function of astrocytes.

## Conclusions

These results collectively support the conclusion that AM provides significant neuroprotection and immunomodulatory effects when applied immediately following SCI. The most critical therapeutic actions- limiting tissue destruction and controlling the formation of the inhibitory glial scar- are supported by the quantitative reduction in cavitation area and the qualitative/quantitative decrease in GFAP expression.

## References

[1] VanderHorst V. G. J.
M., Ulfhake B. (2006). The Organization
of the Brainstem and Spinal Cord of
the Mouse: Relationships between Monoaminergic, Cholinergic, and Spinal
Projection Systems. J. Chem. Neuroanat..

[2] Gibbs K., Beaufort A., Stein A., Leung T. M., Sison C., Bloom O. (2019). Assessment of Pain Symptoms and Quality of Life Using the International
Spinal Cord Injury Data Sets in Persons with Chronic Spinal Cord Injury. Spinal Cord Ser. Cases.

[3] Barbiellini
Amidei C., Salmaso L., Bellio S., Saia M. (2022). Epidemiology
of Traumatic Spinal Cord Injury: A Large Population-Based Study. Spinal Cord.

[4] Johansson E., Luoto T. M., Vainionpää A., Kauppila A. M., Kallinen M., Väärälä E., Koskinen E. (2021). Epidemiology of Traumatic Spinal Cord Injury in Finland. Spinal Cord.

[5] Khadour F. A., Khadour Y. A., Meng L., XinLi C., Xu T. (2024). Epidemiology
Features of Traumatic and Non-Traumatic Spinal Cord Injury in China,
Wuhan. Sci. Rep..

[6] Lund M. C., Ellman D. G., Nissen M., Nielsen P. S., Nielsen P. V., Jørgensen C., Andersen D. C., Gao H., Brambilla R., Degn M., Clausen B. H., Lambertsen K. L. (2022). The Inflammatory
Response after Moderate Contusion Spinal Cord Injury: A Time Study. Biology.

[7] Hachem L. D., Hong J., Velumian A., Mothe A. J., Tator C. H., Fehlings M. G. (2023). Excitotoxic Glutamate Levels Drive Spinal Cord Ependymal
Stem Cell Proliferation and Fate Specification through CP-AMPAR Signaling. Stem Cell Rep..

[8] Jiang T., Qin T., Gao P., Tao Z., Wang X., Wu M., Gu J., Chu B., Zheng Z., Yi J. (2023). SIRT1
Attenuates Blood-Spinal Cord Barrier Disruption after Spinal Cord
Injury by Deacetylating P66Shc. Redox Biol..

[9] You Z., Gao X., Kang X., Yang W., Xiong T., Li Y., Wei F., Zhuang Y., Zhang T., Sun Y., Shen H., Dai J. (2023). Microvascular Endothelial Cells Derived
from Spinal Cord Promote
Spinal Cord Injury Repair. Bioact Mater..

[10] Ma Z., Liu T., Liu L., Pei Y., Wang T., Wang Z., Guan Y., Zhang X., Zhang Y., Chen X. (2024). Epidermal
Neural Crest Stem Cell Conditioned Medium Enhances Spinal Cord Injury
Recovery via PI3K/AKT-Mediated Neuronal Apoptosis Suppression. Neurochem. Res..

[11] Ramadan W. S., Abdel-Hamid G. A., Al-Karim S., Abbas A. T. H. (2017). Immunohistochemical
and Ultrastructural Study of Secondary Compressed Spinal Cord Injury
in a Rat Model. Folia Histochem Cytobiol..

[12] Wanner I. B., Anderson M. A., Song B., Levine J., Fernandez A., Gray-Thompson Z., Ao Y., Sofroniew M. V. (2013). Glial Scar
Borders Are Formed by Newly Proliferated, Elongated Astrocytes That
Interact to Corral Inflammatory and Fibrotic Cells via STAT3-Dependent
Mechanisms after Spinal Cord Injury. J. Neurosci..

[13] dos
Santos Ramalho B., Marques Pestana F., Andrade Prins C., Soares dos Santos Cardoso F., Rufino Cavalcante D., Augusto Lopes de Souza S., Gutfilen B., Martins
de Almeida F., Blanco Martinez A. M. (2019). Effects of Different Doses of Mesenchymal
Stem Cells on Functional Recovery After Compressive Spinal-Cord Injury
in Mice. Neuroscience.

[14] Luijerink L., Rodriguez M., Machaalani R. (2024). Quantifying GFAP Immunohistochemistry
in the Brain – Introduction of the Reactivity Score (R-Score)
and How It Compares to Other Methodologies. J. Neurosci Methods.

[15] Chen X., Chen C., Hao J., Qin R., Qian B., Yang K., Zhang J., Zhang F. (2018). AKR1B1 Upregulation
Contributes to Neuroinflammation and Astrocytes Proliferation by Regulating
the Energy Metabolism in Rat Spinal Cord Injury. Neurochem. Res..

[16] Oberheim N. A., Takano T., Han X., He W., Lin J. H. C., Wang F., Xu Q., Wyatt J. D., Pilcher W., Ojemann J. G., Ransom B. R., Goldman S. A., Nedergaard M. (2009). Uniquely Hominid
Features of Adult Human Astrocytes. J. Neurosci..

[17] Rouach N., Koulakoff A., Abudara V., Willecke K., Giaume C. (2008). Astroglial
Metabolic Networks Sustain Hippocampal Synaptic Transmission. Science.

[18] Takano T., Tian G. F., Peng W., Lou N., Libionka W., Han X., Nedergaard M. (2006). Astrocyte-Mediated
Control of Cerebral Blood Flow. Nat. Neurosci..

[19] Hubscher C.
H., Wyles J., Gallahar A., Johnson K., Willhite A., Harkema S. J., Herrity A. N. (2021). Effect of Different Forms of Activity-Based
Recovery Training on Bladder, Bowel, and Sexual Function After Spinal
Cord Injury. Arch Phys. Med. Rehabil..

[20] Campelo M. B. D., Santos J. D. A. F., Filho A. L. M. M., Ferreira D. C. L., Sant’anna L. B., De Oliveira R. A., Maia L. F., Arisawa E. Â.
L. (2018). Effects of the Application
of the Amniotic Membrane in the Healing Process of Skin Wounds in
Rats. Acta Cir Bras..

[21] Santos J. D. A. F., Nicolau R. A., Sant’anna L. B., Paterno J. C., Cristovam P. C., Santos J. D. M., Arisawa E. (2021). Diabetic Foot
Wounds Treated With
Human Amniotic Membrane and Low-Level Laser Therapy: A Pilot Clinical
Study. Wound Manag. Prev..

[22] Amorim F. C. M., Arisawa E. Â.
L., Sant’anna L. B., Rodrigues A. B. M., Costa D. R. (2023). Preclinical Study of Experimental
Burns Treated with Photobiomodulation and Human Amniotic Membrane,
Both Isolated and Associated. Rev. Lat Am. Enfermagem.

[23] Nicodemo M. D. C., Neves L. R. D., Aguiar J. C., Brito F. D. S., Ferreira I., Sant’anna L. B., Raniero L. J., Martins R. Á.
L., Barja P. R., Arisawa E. A. L. S. (2017). Amniotic Membrane as an Option for
Treatment of Acute Achilles Tendon Injury in Rats. Acta Cir Bras..

[24] Sant’anna L. B., Cargnoni A., Ressel L., Vanosi G., Parolini O. (2011). Amniotic Membrane
Application Reduces Liver Fibrosis in a Bile Duct Ligation Rat Model. Cell Transplant.

[25] Correia D. C. C., de Lima L. B., Sant’anna L. B., Lima M. O., Arisawa E. A. L. S. (2025). Histological
Analysis of Spinal Cord Injury Treated with Amniotic Membrane. Medicina.

[26] Furdova A., Czanner G., Koller J., Vesely P., Furda R., Pridavkova Z. (2023). Amniotic Membrane
Application in Surgical Treatment
of Conjunctival Tumors. Sci. Rep..

[27] Alves A. P. D. S., Teixeira R. J. M., Silva R. M. D., Canevari R. D. A., Sant’anna L. B. (2024). Amniotic Membrane Modulates MMP9
and MMP12 Gene and Protein Expression in Experimental Model of the
Hepatic Fibrosis. An Acad. Bras Cienc..

[28] Tehrani F. A., Modaresifar K., Azizian S., Niknejad H. (2017). Induction
of Antimicrobial
Peptides Secretion by IL-1β Enhances Human Amniotic Membrane
for Regenerative Medicine. Sci. Rep..

[29] Jerman U. D., Veranič P., Kreft M. E. (2014). Amniotic Membrane Scaffolds Enable
the Development of Tissue-Engineered Urothelium with Molecular and
Ultrastructural Properties Comparable to That of Native Urothelium. Tissue Eng. Part C Methods.

[30] DATASUS Relatorio_2014_Datasus. DATASUS, 2014.

[31] Damy S. B., Camargo R. S., Chammas R., Figueiredo L. F. P. (2010). The
Fundamentals of Experiments with Animals-Applications in Experimental
Surgery. Rev. Assoc. Med. Bras..

[32] Gu Y., Cheng X., Huang X., Yuan Y., Qin S., Tan Z., Wang D., Hu X., He C., Su Z. (2019). Conditional
Ablation of Reactive Astrocytes to Dissect Their Roles in Spinal Cord
Injury and Repair. Brain Behav. Immun..

[33] Okada S., Nakamura M., Katoh H., Miyao T., Shimazaki T., Ishii K., Yamane J., Yoshimura A., Iwamoto Y., Toyama Y., Okano H. (2006). Conditional
Ablation
of Stat3 or Socs3 Discloses a Dual Role for Reactive Astrocytes after
Spinal Cord Injury. Nat. Med..

[34] Herrmann J. E., Imura T., Song B., Qi J., Ao Y., Nguyen T. K., Korsak R. A., Takeda K., Akira S., Sofroniew M. V. (2008). STAT3 Is a Critical Regulator of
Astrogliosis and Scar
Formation after Spinal Cord Injury. J. Neurosci..

[35] Chehrehasa F., Cobcroft M., Young Y. W., Mackay-Sim A., Goss B. (2014). An Acute Growth Factor Treatment That Preserves Function after Spinal
Cord Contusion Injury. J. Neurotrauma.

[36] Lu Y., Chen C., Wang H., Du R., Ji J., Xu T., Yang C., Chen X. (2022). Astrocyte-Derived
SEVs Alleviate
Fibrosis and Promote Functional Recovery after Spinal Cord Injury
in Rats. Int. Immunopharmacol..

[37] Coassin M., Lambiase A., Micera A., Tirassa P., Aloe L., Bonini S. (2006). Nerve Growth Factor Modulates in
Vitro the Expression
and Release of TGF-Β1 by Amniotic Membrane. Graefe’s Archive Clin. Exp. Ophthalmol..

[38] Zhou H. L., Zhang X. J., Zhang M. Y., Yan Z. J., Xu Z. M., Xu R. X. (2016). Transplantation
of Human Amniotic Mesenchymal Stem Cells Promotes
Functional Recovery in a Rat Model of Traumatic Spinal Cord Injury. Neurochem. Res..

[39] Hara M., Kobayakawa K., Ohkawa Y., Kumamaru H., Yokota K., Saito T., Kijima K., Yoshizaki S., Harimaya K., Nakashima Y., Okada S. (2017). Interaction of Reactive
Astrocytes with Type I Collagen Induces Astrocytic Scar Formation
through the Integrin–N-Cadherin Pathway after Spinal Cord Injury. Nat. Med..

[40] Moreno S. E., Enwerem-Lackland I., Dreaden K., Massee M., Koob T. J., Harper J. R. (2024). Human Amniotic Membrane Modulates Collagen Production
and Deposition in Vitro. Sci. Rep..

[41] Álvarez Z., Kolberg-Edelbrock A. N., Sasselli I. R., Ortega J. A., Qiu R., Syrgiannis Z., Mirau P. A., Chen F., Chin S. M., Weigand S. (2021). Bioactive Scaffolds with Enhanced Supramolecular Motion
Promote Recovery from Spinal Cord Injury. Science.

[42] Hassan M. P., Abdollahifar M. -A., Aliaghaei A., Tabeie F., Vafaei-Nezhad S., Norouzian M., Abbaszadeh H. A. (2021). Photobiomodulation Therapy Improved
Functional Recovery and Overexpression of Interleukins-10 after Contusion
Spinal Cord Injury in Rats. J. Chem. Neuroanat..

[43] Li X., Zhang C., Haggerty A. E., Yan J., Lan M., Seu M., Yang M., Marlow M. M., Maldonado-Lasunción I., Cho B. (2020). The Effect
of a Nanofiber-Hydrogel Composite on Neural
Tissue Repair and Regeneration in the Contused Spinal Cord. Biomaterials.

[44] Štepánková K., Chudíčková M., Šimková Z., Martinez-Varea N., Kubinová Š., Urdzíková L. M., Jendelová P., Kwok J. C. F. (2023). Low Oral Dose of 4-Methylumbelliferone
Reduces Glial Scar but Is Insufficient to Induce Functional Recovery
after Spinal Cord Injury. Sci. Rep..

[45] Zarei-Kheirabadi M., Hesaraki M., Kiani S., Baharvand H. (2019). In Vivo Conversion
of Rat Astrocytes into Neuronal Cells through Neural Stem Cells in
Injured Spinal Cord with a Single Zinc-Finger Transcription Factor. Stem Cell Res. Ther..

